# Phage Endolysins as an Alternative Biocontrol Strategy for Pathogenic and Spoilage Microorganisms in the Food Industry

**DOI:** 10.3390/v17040564

**Published:** 2025-04-14

**Authors:** Maryoris E. Soto Lopez, Fernando Mendoza-Corvis, Jose Jorge Salgado-Behaine, Ana M. Hernandez-Arteaga, Víctor González-Peña, Andrés M. Burgos-Rivero, Derrick Cortessi, Pedro M. P. Vidigal, Omar Pérez-Sierra

**Affiliations:** 1 Grupo de Investigación en Propiedades y Procesos Alimentarios—GIPPAL, Departamento de Ingeniería de Alimentos, Facultad de Ingeniería, Universidad de Córdoba, Carrera 6 N° 76-103, Código, Montería 230002, Colombia; fmendoza@correo.unicordoba.edu.co (F.M.-C.); josesalgadob@correo.unicordoba.edu.co (J.J.S.-B.); anahernandeza@correo.unicordoba.edu.co (A.M.H.-A.); vgonzalezpena@correo.unicordoba.edu.co (V.G.-P.); aburgosrivero50@correo.unicordoba.edu.co (A.M.B.-R.); oaperez@correo.unicordoba.edu.co (O.P.-S.); 2 Animal and Dairy Sciences Department, University of Wisconsin-Madison (UW-Madison), Madison, WI 53706-1205, USA; dabold@wisc.edu (D.C.); pedro.vidigal@ufv.br (P.M.P.V.); 3 Núcleo de Análise de Biomoléculas (NuBioMol), Campus da UFV, Universidade Federal de Viçosa (UFV), Viçosa 36570-900, MG, Brazil

**Keywords:** bacteriophages, endolysins, biocontrol, antibacterial agents, bacterial lysis, phage enzymes, muralitic activity

## Abstract

Food contamination by pathogenic and spoilage bacteria causes approximately 47 million cases of foodborne diseases in the United States and leads to tons of food spoilage, worsening the food loss situation worldwide. In addition, conventional preservation treatments implemented in the food industry decrease food’s nutritional and organoleptic quality. Therefore, there is a need for new alternatives to counteract food contamination without altering its characteristics. Endolysins are a promising strategy due to their unique properties, such as host specificity, synergism with other antibacterial agents, mode of action, and low probability of resistance development. These characteristics differentiate them from other antibacterial agents used in the food industry. Endolysins are enzymes produced by bacteriophages during the process of bacterial infection and lysis. This review describes the advances related to endolysin application systems in food, considering their potential for food safety and an overview of the application conditions according to the type of food and bacteria to be controlled. We also highlight the need for new studies on endolysin encapsulation and prolongation of the action time in cases of outbreaks that allow obtaining key information to improve the application of endolysins in different food matrices during food processing and storage

## 1. Introduction

Food contamination by pathogenic bacteria is the main cause of foodborne disease (FBD) and a global public health concern [1]. According to the World Health Organization (WHO) report on the global burden of FDB, there are approximately 600 million people become ill due to the consumption of contaminated food, and 420,000 die from these causes [2]. Although the food industry uses physical and chemical methods to control bacterial contamination, these approaches have limitations, including sensory and nutritional alterations, chemical residues, bacterial resistance, and high costs [3,4,5]. Therefore, developing alternative antibacterial agents is crucial. Among the proposed strategies, using bacteriophages and their enzymes has emerged as a promising biocontrol approach to enhance food safety and prevent bacterial proliferation [6,7].

Bacteriophages are viruses that infect bacteria and have demonstrated bactericidal activity in numerous studies, including the biocontrol of *Escherichia coli* (*E. coli*) in milk, ground beef, and spinach [8], *Pseudomonas* spp. in raw milk [9], and *Salmonella* spp. in chicken, pork, lettuce, and milk [10,11]. They have also been used to control *Vibrio parahaemolyticus* contamination in seafood products like fish and shellfish [12,13]. Classified as Generally Recognized As Safe (GRAS) by the US Food and Drug Administration (FDA), phages have been applied as food preservatives in countries such as the United States, Canada, Israel, Switzerland, Australia, and New Zealand, among others [14].

During infection and cell lysis, phages produce enzymes such as virion-associated lysins (VAL), holins, pinholins, and endolysins, which degrade the bacterial cell wall, facilitating phage genetic material injection and subsequent lysis [15]. Among the most studied lytic enzymes, endolysins specifically target bacterial hosts through different catalytic domains that cleave peptidoglycan bonds. Notably, no bacterial resistance to endolysins has been reported, making them a promising tool for controlling pathogenic and spoilage bacteria in food [16,17].

Endolysin applications in the food industry have demonstrated effectiveness against *Staphylococcus aureus*, *Listeria monocytogenes*, *Clostridium perfringens*, and *Bacillus cereus*, among others [18,19,20,21]. Their efficacy is particularly high against *Gram*-positive bacteria due to their exposed peptidoglycan layer [22]. However, the outer membrane (OM) limits endolysin activity in *Gram*-negative bacteria by protecting peptidoglycan [23]. To address this, studies combining endolysins with permeabilizing agents have shown success in controlling *Salmonella* spp., *Pseudomonas aeruginosa*, *Campylobacter jejuni*, and *E. coli* [24,25,26,27,28].

Endolysins can lyse bacteria regardless of their metabolic state, including biofilms on food industry surfaces [15,29]. Additionally, they exhibit synergistic effects when combined with antimicrobial agents, organic acids, or high hydrostatic pressure treatments [24,30,31].

This review aims to analyze the potential progress of endolysins as antimicrobial agents against foodborne pathogenic and spoilage bacteria. Initially, a general description of the characteristics of bacteriophages and their enzymes will be presented. Next, studies on applying these enzymes for the biocontrol of *Gram*-negative and *Gram*-positive bacteria in food will be addressed. Finally, their strengths and limitations will be discussed.

## 2. Bacteriophages and Their Enzymes During the Infection Process

Bacteriophages, or phages, are considered the biosphere’s most abundant and diverse biological entities [32]. Due to their lack of a complete cellular structure, their replication depends on the infection and lysis of a bacterial host. The phage multiplication cycle begins with adsorption through the irreversible binding of receptor-binding proteins (RBPs) to ligands (receptors) on the bacterial surface. Following adsorption, the phage undergoes conformational changes in the baseplate, leading to the contraction of the tail sheath, which punctures the bacterial cytoplasmic membrane. Additionally, phages release virion-associated peptidoglycan hydrolases (VAPGHs), lysozymes, and other proteins present in the needle sheath domains that assist in degrading the peptidoglycan cell wall [15].

The infection process continues with the injection of genetic material into the bacterial cytoplasm through the pores created in the bacterial membrane [33]. Once the phage genetic material has entered the host cell, different multiplication cycles can occur, with the most common being lytic, lysogenic, chronic, and pseudolysogenic cycle [34,35]. Among these, the lytic cycle is the most relevant for applications, as it leads to the destruction of the host cell.

**Figure 1:**
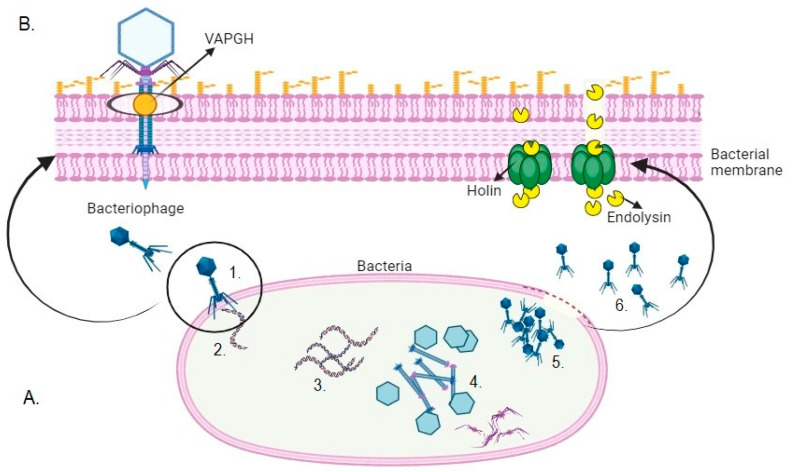
(**A**) Lytic cycle: (1) phage attachment to the bacterium; (2) injection of genetic material; (3) replication of the phage genome; (4) production of phage protein structures; (5) phage assembly; (6) lysis and release of virions. (**B**) Activity of the enzymes VAPGH, endolysins, and holins. Adapted from Gutiérrez et al. [41].

During the lytic cycle (see Figure 1A), phages hijack bacterial metabolism, taking control of the biosynthetic machinery for mRNA translation, nucleic acid replication, and structural protein synthesis [36] Subsequently, new virions are assembled, with the replicated genetic material packaged into the capsid, followed by the attachment of neck proteins and the assembly of the phage tail [37,38]. At the end of the cycle, the release of virions is facilitated by activating holin and endolysin enzymes, which work together to degrade the bacterial cell wall. Holins penetrate the inner membrane, forming pores that allow endolysins to access the peptidoglycan layer, leading to cell wall rupture and bacterial lysis (see Figure 1B) [39,40].

## 3. Phage Enzymes

Enzymes produced by bacteriophages are highly efficient molecules that target the integrity of the bacterial cell wall, producing cell lysis and release of viral progeny [16,29]. During infection and cell lysis, different enzymes such as VAPGHs, holins, pinholins, and endolysins are involved, whose main function is to target the peptidoglycan bonds of the bacterial cell wall [42,43]. 

VAPGHs, also called lytic structural proteins [44], are phage-encoded enzymes with a virion-binding domain and one or two lytic domains responsible for perforating the peptidoglycan from outside the bacterial cell wall. By creating a small hole in the cell envelope, the phage facilitates the introduction of its tail tube, allowing the injection of the viral genome, thereby initiating the infection process (Figure 1B) [45].

Phages with double-stranded DNA genomes accelerate bacterial lysis and the release of virions by activating different phage enzymes that act from within the host cell. Initially, they permeabilize the inner membrane, degrade the peptidoglycan of the cell wall, and ultimately destabilize the cellular structure until lysis occurs. The enzymes that participate in this process are known as holins, pinholins, and endolysins [15,23,46,47].

Holins are small hydrophobic enzymes activated by phages to be embedded in the cytoplasmic membrane and form pores or holes. They establish the start of cell lysis by providing a path through which lysins cross, allowing them to reach the peptidoglycan matrix (Figure 1B) [15]. Sometimes, the pores produced by holins are not large enough to allow the passage of lysins, but they do allow the entry of ions that change the membrane potential and thus activate the action of lysins [48].

Pinholins are a type of phage-encoded holin responsible for forming small nanometric-sized pores (<2 nm in diameter), through which protons pass that help depolarize the membrane, allowing the non-specific passage of a folded protein. Based on this, for the bacterial lysis process to take place, pinholins require SAR endolysins, which have an N-terminal transmembrane domain that activates the secondary translocon, resulting in the transport of the membrane-bound endolysin in an inactive form [47,49].

Endolysins or lysins are enzymes encoded by phages in the bacterial cytoplasm at the end of the infection process, where they are harmless to the bacteria as long as they do not manage to reach the peptidoglycan in their active form [50]. As for their classification, endolysins are divided into five classes according to their mechanism of action on peptidoglycan, including (I) lysozymes or muramidases (N-acetyl-β-d-muramidases), (II) lytic transglycosylases, (III) N-acetyl-muramoylamidases, (IV) N-acetyl-β-d-glucosaminidases, and (V) endopeptidases. Endolysins are highly specific for peptidoglycan, exhibit a broad lytic spectrum, induce lower bacterial resistance, and do not disrupt the native microbiota of foods [51]. 

Endolysins are responsible for cleaving the peptidoglycan of the cell wall from the inside, a process essential for achieving rapid and efficient bacteriolysis [16,46]. This process produces an osmotic imbalance due to the high internal pressure of the bacterial cell and leads to the rupture of the cell membrane layer, resulting in the extrusion of the cytoplasmic membrane and the final hypotonic lysis that facilitates the release of the newly replicated phages [52]. Endolysins generally cannot reach the cell wall in an active state on their own, so they require the activity of other phage enzymes to achieve this goal, as is the case of holins and pinholins that act directly on the cytoplasmic membrane [23,39].

**Figure 2:**
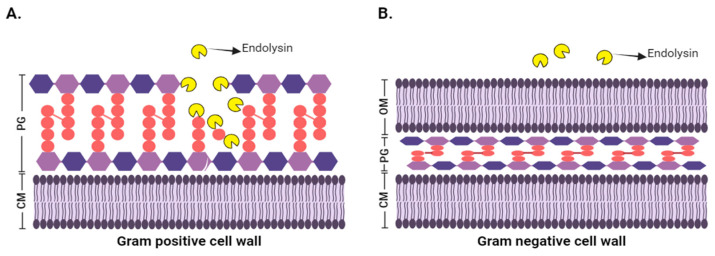
Exogenous application of an endolysin. (**A**) Application to a *Gram*-positive cell: The endolysin binds to the peptidoglycan and cleaves the peptidoglycan bonds, leading to cell lysis. (**B**) Application to a *Gram*-negative cell: The outer membrane (OM) acts as a selective barrier that protects the peptidoglycan.

In *Gram*-positive bacteria, due to the absence of an OM, the peptidoglycan is exposed to endolysins’ action when added exogenously. Thus, lysins act on the peptidoglycan and break the bacterial membrane, producing lysis of the bacteria (see Figure 2A). Therefore, lysins are considered specific and effective antimicrobials that can lyse resistant cells and prevent the generation of resistant mutants [53,54]. Lysins targeting *Gram*-positive bacteria exhibit a modular architecture, where the catalytic function and specific cell wall recognition are separated into two or more functional domains; i.e., lysins contain an Enzymatically Active Domain (EAD) at the N-terminus, for specific cleavage of covalent bonds in the peptidoglycan backbone of the host cell wall, and a Cell Wall Binding ssDomain (CBD) at the C-terminus, for substrate recognition and specific binding to the host cell wall [55,56,57,58].

*Gram*-negative bacteria, in turn, possess an OM that protects the peptidoglycan from external factors, making it impermeable and preventing enzyme activity (see Figure 2B) [59]. In this case, most endolysins targeting *Gram*-negative bacteria exhibit a catalytic function restricted to a single catalytic globular domain, meaning they contain only an EAD and rarely exhibit a modular structure [30].

Table 1 lists various studies demonstrating the strong potential of endolysins to inhibit the growth of foodborne pathogenic bacteria. These studies show substantial decreases in bacterial populations and suggest their potential as a promising alternative in the food industry.

## 4. Strategies for Mining and Identifying Phage Endolysins

Endolysins and other phage enzyme screening has advanced significantly due to improvements in bioinformatics and artificial intelligence (AI) algorithms. Tools such as BLAST [66] and DIAMOND [67], which rely on sequence-alignment-based comparisons, have facilitated the annotation of endolysins across diverse genomic and metagenomic datasets. These tools detect significant alignments with putative homologs, enabling the identification of endolysins with similarities to those in reference databases. Additionally, rule-based pipelines incorporating predictive tools for key features of the multi-domain architecture of phage endolysins have expanded the repertoire of these enzymes, enriching reference datasets. Endolysins encoded by bacteriophages infecting *Gram*-positive bacteria contain at least one catalytic domain and one or more cell wall-binding domains [68].

In contrast, those infecting *Gram*-negative bacteria often encode endolysins with a single catalytic domain combined with N-terminal transmembrane regions, such as signal-arrest-release (SAR) domains [69], or -terminal transmembrane helices [70]. Rule-based pipelines leverage tools like HMMER (http://hmmer.org/, accessed on 1 March 2025) and InterProScan [71] to identify domain regions, alongside SignalP [72] for detecting N-terminal signal peptides and DeepTMHMM [73] for predicting transmembrane regions. More recently, machine learning algorithms, driven by the growing influence of artificial intelligence (AI), have been employed to develop predictive tools for endolysin sequences. The tools include Lypred [74], which uses support vector machine (SVM) methods to classify protein pseudo-amino acid composition (PseAAC) features; CWLypred [57], an extension of Lypred that incorporates a broader set of features; and DeepMineLys [75], which uses a convolutional neural network (CNN) with a two-track architecture of features [69]. These AI models enhance the identification of endolysins among hypothetical proteins that diverge significantly from reference databases due to the high diversity of bacteriophages.

Using DeepMineLys, Fu et al. [75] mined 370,849 proteins retrieved from human microbiome datasets and identified 18,514 endolysins, 20,340 virion-associated lysins (VALs), and 6,784 holins. From these, they selected 15 lysin candidates predicted to target *Gram*-positive or *Gram*-negative bacteria based on their top-ranking prediction scores, along with one additional candidate exhibiting an unusual architecture—it lacked a cell wall binding domain (CBD) and contained only an enzymatically active domain (EAD). These candidates were heterologously expressed and experimentally validated for muralytic activity by assessing their ability to degrade the peptidoglycan layer of *P. aeruginosa* PAO1. A commercial lysozyme derived from hen egg white (HEWL) was a benchmark. Remarkably, some among selected lysins demonstrated higher activity than HEWL, with enzymatic activity ranging from 1.3- to 6.2-fold greater than the benchmark.

## 5. Application of Phage Enzymes in Foods for the Control of *Gram*-Positive Bacteria

### 5.1. Enzymes for the Control of Staphylococcus aureus

*Staphylococcus aureus* (*S. aureus*) is a *Gram*-positive bacterium present in the microbiota of the skin and mucosa of humans and animals, in the environment, and commonly in raw milk from cows with mastitis [76]. *S. aureus* is one of the main agents responsible for FBD outbreaks in humans due to its ability to form biofilms, develop resistance to antibiotics such as methicillin and vancomycin, and produce heat-stable enterotoxins that can withstand heat treatments during the food production process [77]. The foods most frequently implicated in staphylococcal food poisoning include milk, certain cheeses, meat products, and vegetables, with transmission occurring through food handlers, by cross-contamination, or by diseases such as mastitis [60,78,79].

In products such as milk, meats, and vegetables, the application of endolysins for the biocontrol of *S. aureus* has been investigated. In general, the endolysins used to target *S. aureus* contain three domains: a catalytic CHAP (cysteine, histidine-dependent amidohydrolases/peptidases) domain at the N-terminal end, a central N-acetylmuramoyl-L-alanine amidase domain, and a C-terminal cell wall-binding domain (CBD) [80]. The CHAP domain is essential for degrading amide bonds between N-acetylmuramic acid and L-alanine in the peptidoglycan of the *S. aureus* cell wall [78]. Regarding the amidase domain, some studies suggest that it enhances the affinity of the CBD domain [55]. However, other studies have reported that removing the amidase domain improves the anti-staphylococcal activity of the endolysin LysRODI [41,53].

Endolysins used against *S. aureus* have demonstrated efficient activity at room temperature (25 °C–7 °C), near-neutral pH, in the presence of calcium ions required by the CHAP domain, and moderate salt concentrations (0–2%). Milk and cheese maintain these conditions, making endolysins promising candidates for food safety applications. Park et al. [81] found that the application of 2 μM of the recombinant endolysin LysSAP27 reduced the growth of *S. aureus* (ATCC 6538) by 3.36 log_10_ CFU/mL in contaminated pasteurized milk (10^5^ CFU/mL) after 2 h of incubation at 25 °C. Similarly, Chang et al. [82] evaluated the anti-staphylococcal activity of endolysin LysSA11, derived from phage SA11, applied at concentrations of 0, 1.125, 2.25, 3.375, 4.5, and 9 μM in commercial pasteurized milk contaminated with *S. aureus* (2 × 10^5^ CFU/mL). Significant inhibitory effects were observed starting at 3.375 μM, with the enzyme functioning more effectively at 25 °C. At a concentration of 9 μM, viable cells were reduced to undetectable levels after 30 and 60 min of treatment.

Other approaches to enhance the inhibitory effect of endolysins are also being explored. Genetic engineering has been proposed to modify these enzymes’ domains to improve their binding capacity, solubility, and lytic activity. Son et al. [55] performed random domain swapping among four *S. aureus* endolysins (LysSA12, LysSA97, LysSA11, and LysSAP4), resulting in a chimeric endolysin named Lys109. Lys109 consists of a CHAP domain from LysSA12 at the N-terminal region, an amidase domain from LysSA97 in the central region, and CBD from LysSA97 at the C-terminal region. Furthermore, the improved endolysin Lys109 (0.9 μM) exhibited a superior inhibitory effect compared to the original endolysin (LysSA12) when applied to pasteurized commercial milk and a stainless-steel surface contaminated with *S. aureus* CCARM 3090 (10^5^ CFU/mL) at 25 °C. The results showed that *S. aureus* was reduced to undetectable levels after 45 min of treatment with Lys109, whereas no significant reductions were observed with LysSA12. These findings highlight that the N-terminal CHAP domain is essential for *S. aureus* lysis and that the amidase domain in the central region can enhance endolysin activity.

On the other hand, Agún et al. [53] compared the lytic effect of the recombinant endolysin LysRODIΔAmi (4 μM) and its original form (LysRODI (4 μM)) in three commercial milks (UHT skimmed, UHT whole and pasteurized whole) contaminated with *S. aureus* Sa9 (10^3^, 10^4^ and 10^5^ CFU/mL). LysRODIΔAmi reduced the viable cell count below the detection limit (100 CFU/mL) in almost all treated samples after 2 h at 37 °C, while the inhibitory effect with the original endolysin was moderate. In the study, it was evident that the control of the bacterial population with endolysin was limited because the contaminating population of *S. aureus* treated with LysRODIΔAmi showed regrowth within hours of treatment in UHT milk. These results indicate that although there have been successful cases with the genetic improvement of endolysins, there are challenges related to their application, for example, improving their action at low temperatures, prolonging the biocontrol effect over time, and reducing the costs related to their production regarding their purification and recombination, considering that although the modified endolysin LysRODIΔAmi had a more significant effect, its associated cost was high [83].

Based on the above, Youssef et al. [83] evaluated the biocontrol effect of different combinations of LysRODIΔAmi (0.0075, 0.015, 0.06, or 0.12 μM), phage phiIPLA-RODI (10^7^ PFU/mL) and nisin (1.5 μg/mL) in the biocontrol of *S. aureus* (10^5^ CFU/mL) during fresh cheese production, using two concentrations of calcium chloride (CaCl_2_, 0.02 or 0.2% *w*/*v*), by enzymatic coagulation of milk at 32 °C for two hours. The highest antibacterial activity was observed when 0.2% CaCl_2_ with 0.06 or 0.12 μM of LysRODIΔAmi was applied, with a viable cell count below the detection limit (100 CFU/mL) during milk coagulation. However, future studies need to identify the minimum concentration of CaCl_2_ that achieves this biocontrol effect while avoiding alterations in the physicochemical and organoleptic characteristics of the cheese.

On the other hand, the cheese obtained in the study of Youssef et al. [83] was stored at 4 or 12 °C for 14 days to evaluate the anti-staphylococcal activity of the different combinations (CaCl_2_, phages, nisin, and LysRODIΔAmi). In most of the combinations studied, there was a greater reduction in the bacterial population compared to individual effects, considering that this effect was additive and not synergistic. The results obtained from the biocontrol of *S. aureus* during the 14 days of storage at 4 °C, in the treatment of 0.02% CaCl_2_ and a combination of phages, nisin and LysRODIΔAmi 0.12 μM, showed biocontrol of bacterial contamination below the detection limit; however, at 12 °C, the treatment that included the three antimicrobials presented lower activity, providing an average reduction of 3.70 and 4.10 log_10_ CFU/mL with CaCl_2_ at 0.2% and 0.02%, respectively. In this study, it was found that the combination used and the increase in the concentration of CaCl_2_ are treatments that can reduce the amount of endolysin required for the biocontrol of *S. aureus* in cheese making, allowing for cost reduction.

Another study also reported the construction of a constitutive secretory vector to deliver the recombinant endolysin Lysdb from the phage phiLdb into *Lactobacillus casei* BL23 to evaluate the antibacterial activity of Lysdb in controlling the growth of *S. aureus* (ATCC 33591, 4 × 10^4^ CFU/mL) in cheese making. In the control samples for milk and cheese, the *S. aureus* count remained at approximately 10^9^ and 10^7^ CFU/g, respectively, remaining constant until the end of fermentation. In contrast, the samples treated with Lysdb were reduced to 10^4^ CFU/mL at 360 min, and during the ripening of the experimental cheese stored at 10 °C, it decreased to 2.7 × 10^3^ CFU/g after 6 weeks. This study achieved the efficient delivery of Lysdb from a modified *L. casei* strain, allowing biocontrol of *S. aureus* during cheese production and ripening [60].

Endolysins for *S. aureus* have been applied to other foods and contact surfaces. Endolysin LysSA11 was applied to ham, showing significant reductions starting from concentrations of 1.125 μM at 4 and 25 °C in less than one hour of application. This same endolysin was also used for the disinfection of surfaces contaminated with 10^5^ CFU/mL of *S. aureus* MRSA by applying 0.9 and 1.35 μM LysSA11 on stainless steel and polypropylene surfaces where complete elimination of the bacteria was found in 30 min at 25 °C [78]. Under similar conditions, Son et al. [55] studied the disinfection of steel surfaces with the chimeric endolysin Lys109 at 0.1 μM, achieving a reduction below the detection limit in 60 min at 25 °C.

Studies have been conducted on endolysins, combining their activity with other antibacterial agents. Chang et al. [78] evaluated the individual and synergistic effect of phage-derived endolysin LysSA97 (1.88 μM) with the activity of carvacrol (6.66 mM), a compound extracted from essential oils, to control contamination caused by *S. aureus* (10^5^ CFU/mL) in whole and skimmed milk. The results indicated reductions below 2 log_10_ CFU/mL in skimmed milk after 3 h of incubation at 25 °C in the individual application of both antibacterial agents. Under the same conditions, the combination of these agents showed a synergistic effect, reducing the population below the detection limit. However, in the treatment carried out in pasteurized whole milk, there was no synergistic activity. This result is possibly due to the fact that carvacrol is not effectively directed to the bacterial cytoplasmic membrane due to lipids. Therefore, these results suggest that the cocktail of endolysin and carvacrol can be used to control staphylococcal cells in low-fat food products.

Similarly, Chang et al. [78] also evaluated the synergistic effectiveness of LysSA97 endolysin and carvacrol in beef cubes (fat < 10%) contaminated with a suspension of 10^5^ CFU/mL of *S. aureus.* The results indicate that with the application of 30 μL of a solution of 18.8 μM LysSA97 and 6.66 mM carvacrol, a reduction of 2.1 log_10_ CFU/cm^2^ in 3 h was achieved in this product.

### 5.2. Enzymes for the Control of Listeria monocytogenes

*Listeria monocytogenes* (*L. monocytogenes*) is a foodborne pathogen found in soil, water, and animals’ digestive tracts. It is associated with the contamination of ready-to-eat meat products such as sausages, smoked salmon, raw fermented meat sausages, and dairy products such as soft cheeses, unpasteurized milk, and ice cream. It can also be found in fresh fruits and vegetables. Consumption of food contaminated with *L. monocytogenes* can cause listeriosis, one of the most serious foodborne diseases [84].

Furthermore, exogenously applied endolysins can hydrolyze the cell wall of *Gram*-positive bacteria, such as *L. monocytogenes* [19]. Recently, different research projects have been developed on applying endolysins to inhibit *L. monocytogenes* contamination. Among these, Van Nassau et al. [85] conducted an inactivation study of *L. monocytogenes* (10^7^ CFU/mL) using a combination of the endolysins PlyP40, Ply511, or PlyP825 with High Hydrostatic Pressures (HHP). The reported results indicate that, when using a lysin concentration of 0.16 µg/mL and 300 MPa of pressure, the individual effect was reduced by approximately 0.2 and 0.3 log_10_ CFU/mL, respectively. The combined treatments reduced the viable cell count by 5.2, 4.7, and 5.5 log_10_ CFU/mL when HHP was applied with PlyP40, Ply511, or PlyP825, respectively, demonstrating a synergistic effect between the two treatments.

In a similar study, Misiou et al. [86] evaluated the combined effect of the activity of phage-derived endolysin PlyP825 (34 µg/mL) and HHP. They were applied to different foods (milk, mozzarella cheese, and smoked salmon) contaminated with a cocktail of five strains of *L. monocytogenes* (10^7^ CFU/mL) and incubated at 25 °C for 4 h. The results showed a reduction of the *Listeria* population of 4 to 5 log_10_ CFU/mL at a pressure of 400 MPa in milk and mozzarella cheese. However, a higher HHP (500 MPa) was needed to reduce 1.6 log_10_ CFU/mL in the case of smoked salmon. Therefore, the characteristics of the environment or food matrix influence the treatment’s effectiveness. Specifically, the treatment is more effective in liquid products than in solids due to the better distribution of the antimicrobial agent in the medium, ensuring more effective bacterial control.

Studies carried out by Van Nassau et al. [85], and Misiou et al. [86] determined that the increased sensitivity of *L. monocytogenes* cells to pressure is due to the enzymatic degradation of peptidoglycan, the stabilizing layer of the bacterial cell wall, which makes the cell more susceptible to stressors such as HHP, affecting the rigidity and integrity of the cell membrane. The authors verified the synergistic effect between lysins and HHP in their in vitro application. They identified the possibility of reducing pressure levels to biocontrol undesirable bacteria in sensitive foods.

Ibarra-Sánchez et al. [87] evaluated the synergistic activity of recombinant endolysin PlyP100 with nisin when incorporated into the preparation of fresh cheese to control the growth of *L. monocytogenes* (4.2 log_10_ CFU/g). Nisin (250 μg/g) was added to the milk, and endolysin PlyP100 (2.5 or 10 U/g) was added to the curd. The results indicated that after 28 days of incubation at 4 °C, the bacterial population was reduced below the detection limit in all the combinations of nisin and PlyP100 tested. It indicates that the synergy between these two antibacterial agents can control the contamination of *L. monocytogenes*, effectively preventing the growth and recovery of this bacterium in fresh cheese. This study also suggested that PlyP100 or nisin individually do not guarantee the safety of fresh cheese. However, the potential synergy of these bactericides helps to overcome these limitations.

### 5.3. Enzymes for the Control of Clostridium perfringens

*Clostridium perfringens* (*C. perfringens*) is a *Gram*-positive, rod-shaped, spore-forming anaerobic bacterium widely distributed in nature, especially in soil, wastewater, and the intestinal tract of humans and animals [24,88]. Due to its type A enterotoxin, *C. perfringens* produces more than 17 potent toxins and is one of the causes of food poisoning such as gas gangrene and necrotic enteritis in both humans and animals [89,90,91].

Cho et al. [90] obtained a new recombinant endolysin (LysCPAS15) in the genome of the phage CPAS-15 targeting *C. perfringens*. To determine its lytic activity, they applied a concentration of 10 µg/mL of lysin in milk contaminated with *C. perfringens* KCTC 3269 and incubated it at 37 °C for 120 min. The results indicated a 4 log_10_ CFU/mL reduction compared to the control without lysin. Subsequently, the CBD region (LysCPAS15_CBD) of the enzyme was fused to an enhanced green fluorescent protein (EGFP) (EGFP_LysCPAS15_CBD) for evaluating biocontrol and rapid detection of *C. perfringens* KCTC 3269 (10^5^ CFU/mL) in sterilized whole milk, incubated at 37 °C for 1 h. Then, purified LysCPAS15 (10.2 µg/mL) was added and incubated at 37 °C for 0, 30, 60, and 120 min. On the other hand, to evaluate the activity of EGFP_LysCPAS15_CBD (30 µg/mL) in contaminated milk, it was incubated at 37 °C for 5 min. The results showed 4 log_10_ CFU/mL reductions and detection within 5 min. The stability of endolysin was maintained from −20 °C and 40 °C and pH range 4 to 8. However, the action began to decrease at temperatures above 50 °C and pH levels below 2 and above 10, indicating that it is useful for applications at low temperatures and neutral pH. This makes it suitable to apply LysCPAS15 and EGFP_LysCPAS15_CBD in the bioconservation of milk and similar products.

Zhao et al. [92] characterized and evaluated the efficacy of recombinant endolysin cpp-lys, encoded by phage cpp, for the biocontrol of *C. perfringens* J1 (toxin type A) in lettuce. The experiment involved applying 100 μL of *C. perfringens* J1 suspension (∼1 × 10^7^ CFU/cm^2^) to 1 cm^2^ pieces of previously sterilized lettuce, followed by a 60-min drying period. Subsequently, 100 μL of endolysin cpp-lys (10 μg) was evenly distributed on the lettuce surface and incubated anaerobically at 42 °C. After 15, 30, and 60 min, the lettuce samples were transferred to sterile bags containing 20 mL of sterile distilled water and homogenized. The results indicated that the enzyme eliminated more than 4 log_10_ CFU/cm^2^ of the host bacteria within 15 min. Furthermore, cpp-lys remained stable within a temperature range of 4 to 50 °C and a pH range from 4 to 9. Additionally, its storage stability was unaffected after 30 days at temperatures between −80 °C and 25 °C; however, activity decreased by 40% and 18% at 42 °C and 37 °C, respectively. 

Lu et al. [93] evaluated the biocontrol effect of endolysin LysCP28 (5–500 µg/mL), encoded by phage BG3P, against *C. perfringens* strain ATCC 13124 (2.3 × 10^3^ CFU/g) in duck meat stored at 4 °C. The results indicated that 500 µg/mL of LysCP28 reduced *C. perfringens* contamination by more than 3.4 log CFU/g compared to the control sample after 72 h. The bacteriolytic activity of LysCP28 was found to be dose-dependent. Even at low doses, the viable cell count was significantly reduced by 3.2 and 3.08 log CFU/g with 100 µg/mL and 50 µg/mL of LysCP28, respectively, after 72 h.

Choi et al. [7] engineered a chimeric endolysin by combining the EADs of LysCPD9 with various CBDs from the thermostable endolysin LysCPS2 of phage CPS2, which is specific to *C. perfringens*. Among the constructed endolysins, the chimeric endolysin ClyY was selected due to its enhanced binding and lytic activity compared to the parental endolysin LysCPD9. Furthermore, ClyY remained stable across a broad range of NaCl concentrations (0–1000 nM), pH levels (5–9), and temperatures (up to 95 °C). The lytic activity of LysCPD9 and ClyY (1.25 µM) against *C. perfringens* strain ATCC 13124 (10^6^ CFU/mL) was evaluated in milk and beef incubated for 2 h at 25 °C. The results showed that treatment with LysCPD9 (1.25 µM) reduced viable cell count of 1 log_10_ CFU/mL in milk and 1 log_10_ CFU/cm^2^ in meat. However, ClyY at the same concentration reduced 5 log_10_ CFU/mL in milk and 3 log_10_ CFU/cm^2^ in meat. The improvement in lytic activity may be attributed to increased cell wall binding, as ClyY exhibited threefold higher binding activity than LysCPD9.

In a study conducted by Noor Mohammadi et al. [94], the antibacterial capacity of the recombinant endolysin LysCPQ7, derived from the phage CPQ7, was evaluated for the biocontrol of *C. perfringens* in milk and cheese slices incubated at 8 and 24 °C for 24 h. The results indicated reductions in viable *C. perfringens* counts of 1.05 and 1.73 log_10_ CFU/mL in milk at 8 and 24 °C, respectively. LysCPQ7 reduced bacterial viable counts on cheese slices by 4 log_10_ CFU/slice at 8 °C and by more than 4.5 log_10_ CFU/slice at 24 °C.

## 6. Application of Phage Enzymes in Foods for the Control of *Gram*-Negative Bacteria

**Figure 3:**
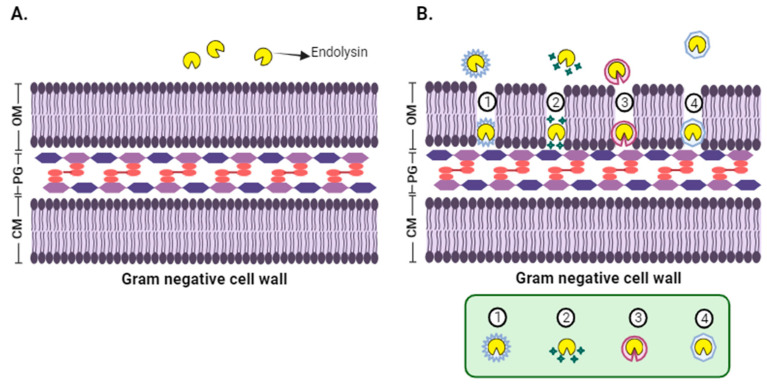
Methods to help endolysins access the OM of *Gram*-negative bacteria: (**A**) *Gram*-negative bacteria possess an OM that acts as a barrier, preventing direct contact with natural endolysins when externally applied. This barrier prevents peptidoglycan degradation and cell lysis. (**B**) (1) Identification of lysins with intrinsic capacity for OM penetration; (2) Endolysin in combination with OMPs; (3) Lysins modified by protein engineering (4) Endolysins in OM-penetrating carrier systems.

*Gram*-negative bacteria possess an OM that protects the peptidoglycan layer, preventing endolysins from accessing the bacterial cell (see Figure 3A) [70]. Recent research has focused on developing strategies to enable the passage of endolysins through the OM of *Gram*-negative bacteria. Accordingly, some lysins have been reported to possess an intrinsic ability to penetrate the OM. The most common feature is the presence of a cationic peptide at the C- or N-terminus that can perturb the OM, giving access to peptidoglycan [95]. Furthermore, several methods have been proposed to help endolysins access the OM, including a synergistic combination with outer membrane permeabilizers (OMPs), such as ethylenediaminetetraacetic acid (EDTA) [96]. EDTA has been successfully used as an OMP for *Gram*-negative bacteria, applied in the genera *Salmonella*, *Escherichia*, and *Pseudomonas* [97,98]. Another alternative is the modification of endolysins by protein engineering to create innolysins, lysocins, and artilysins, which can cross the OM [23,56,96,99,100], as well as the encapsulation of lysins in carrier systems with OM penetration properties (see Figure 3B) [99].

The activity of endolysins on *Gram*-negative bacteria has been evaluated in combination with OMP agents on several occasions [97,100,101,102]. For example, Jiang et al. [100] reported the expression of the endolysin LysSP1 from the phage SLMP1 in *E. coli* and analyzed its lytic characteristics against the *Salmonella enterica* subsp. *enterica serovar* Typhimurium *(S. typhimurium*, ATCC 14028). They pretreated *S. typhimurium* with EDTA (5 mM/L) to evaluate its lytic activity to permeabilize the OM before adding LysSP1 (10 µg). The results showed a synergistic effect between EDTA and the bactericidal activity of LysSP1, resulting in a 3.2 log_10_ CFU/mL reduction in viable *S. typhimurium* cells.

Similarly, Ding et al. [103] identified a novel recombinant endolysin from phage LPSE1, named LysSE24, which exhibits N-acetylmuramidase activity. They evaluated the lytic activity of this enzyme by turbidity reduction assays, combining LysSE24 with OMPs (EDTA at 0.5 mM, citric acid (CA) at 2 mM, or Triton X-100 at 0.1% (*v*/*v*)) in the growth control of *Salmonella enterica* subsp. *enterica serovar* Enteritidis (ATCC 13076) (*S. Enteritidis*). Bacterial strains were pretreated with chloroform (0.5% (*v*/*v*)) for 20 min to destabilize the OM, washed with sterile deionized water, and resuspended in the OMP. The suspension was then mixed with 50 μL LysSE24 (100 nM). The highest antibacterial effect was observed in combining endolysin with Triton X-100, achieving a lytic rate above 75%.

Building on the application of endolysins in combination with OMPs, Baliga et al. [104] investigated the antibacterial activity of recombinant endolysin LysE against *Aeromonas hydrophila* ATCC 7966 (10^6^ CFU/mL) in combination with EDTA as OMP. In this experiment, bacteria were treated with LysE (2 mg/mL) and EDTA (5 mM). A significant reduction in planktonic *A. hydrophila* cells was observed after 30 min of incubation at 30 °C.

Among the studies carried out to control food contamination with *Gram*-negative bacteria by applying phage enzymes and OMPs, one notable example is the study conducted by Nie et al. [30]. They applied 30 µg/mL of EDTA-2Na combined with the endolysin Salmcide-p1, derived from the virulent *Salmonella* phage fmb-p1, at concentrations of 112 µg/mL and 448 µg/mL to evaluate its effect on controlling the contamination of bacterial strains *S. typhimurium* (CMCC 50115) and *Shigella flexneri* (CMCC 51571, 10^4^ CFU/mL) in UHT skimmed milk, stored at 25 or 4 °C. The results showed an approximate reduction of 1.5 log_10_ CFU/mL in bacterial counts at 25 °C after 24 h for both concentrations of Salmcide-p1. The application of the permeabilizer enabled endolysin to penetrate the OM of the bacteria, reaching the peptidoglycan layer of the cell wall and exerting lytic activity.

In a similar study, Zhang et al. [97] used 5 mM EDTA combined with 100 µg/mL of the recombinant endolysin LysSTG2, encoded by the bacteriophage STG2, to control the growth of *Pseudomonas aeruginosa* and *Pseudomonas putida* in different food matrices (bottled water, milk, chicken breast, and salmon) stored at 8, 25 and 37 °C. In bottled water, they observed a 2.2 log_10_ CFU/mL reduction for *P. aeruginosa* and a reduction below the detection limit for *P. putida*; however, no significant effect was observed in milk. The most effective chicken breast treatment involved adding 1 mg/mL of LysSTG2 at 8 °C, resulting in a significant reduction (*p* < 0.05) in bacterial growth. In the salmon sample, 1 mg/mL of LysSTG2 significantly reduced the viability of *P. aeruginosa* and *P. putida* by 0.7 and 1.2 log_10_ CFU per piece, respectively, after 2 h of incubation.

In the study by Shen et al. [16], they cloned and characterized the endolysin LysJNo1 from the phage JNo1. The endolysin was recombinantly expressed, and its lytic activity was evaluated in lettuce artificially contaminated with *E. coli* O157:H7. Subsequently, a combination of EDTA and the recombined endolysin rLysJNo1 (60 µg/mL) was applied. The results showed a 97.8% reduction in *E. coli* O157:H7 after incubation at 25 °C for 60 min, indicating a synergistic interaction between rLysJNo1 and EDTA. This combination has been proposed as a promising strategy for the biocontrol of *E. coli* O157:H7 in lettuce. These studies demonstrate the synergistic effect of endolysins combined with EDTA in the biocontrol of *Gram*-negative bacteria in food. However, further research is needed to assess the efficacy of this combination in different types of foods, considering variables such as temperature, pH, NaCl concentration, ultraviolet light exposure, incubation time, and optimal application concentrations.

Some essential oils, such as carvacrol, eugenol, and thymol, contain high levels of phenolic compounds, which can disrupt the OM of *Gram*-negative bacteria. In the study conducted by Kim et al. [102] the antibacterial effect of these essential oils—allyl isothiocyanate (AITC, 3.0 mg/g), carvacrol (1.5 mg/g), eugenol (1.4 mg/g), or thymol (0.7 mg/g)—was evaluated both individually and in combination with the endolysin LysPB32 (100 μg/g), to control the contamination of *S. typhimurium* (KCCM 40253, 3 × 10^4^ CFU/g) in cooked ground beef. The results showed an enhanced antimicrobial effect when essential oils were combined with LysPB32, with the carvacrol–LysPB32 and eugenol–LysPB32 combinations exhibiting the strongest synergistic effect, resulting in significant reductions in viable cells by 2.3 and 2.5 log_10_ CFU/g, respectively, compared to the control after 24 h of incubation at 37 °C. During seven days of storage at 4 °C, the greatest antibacterial effect was observed for the eugenol–LysPB32 combination (reduction > 2 log_10_ CFU/g), followed by carvacrol–LysPB32 and thymol–LysPB32.

In the study conducted by Zampara et al. [27], an antibacterial agent was designed by fusing a recombinant endolysin with a phage RBP, resulting in Innolysin Cj1, aimed at controlling the growth of *Campylobacter jejuni* (CAMSA 2147). To evaluate its lytic activity, chicken skin was artificially contaminated with 20 μL of a suspension containing 10^4^ CFU/mL of the target bacteria and incubated at 5 °C. The results showed a 1.63 ± 0.46 log_10_ CFU/g reduction after applying 50 μL of Innolysin Cj1.

The antimicrobial activity of endolysins against *Gram*-negative bacteria has also been assessed in combination with antibiotics. Kim et al. [101] investigated the effect of the *Salmonella* phage-encoded endolysin LysPB32 in combination with polymyxins, which enhance the permeability of the OM of *Gram*-negative bacteria. The muralytic activity of LysPB32, polymyxin B, and their combination was evaluated against three strains of *S. typhimurium*. The greatest reduction—over 7 log_10_ CFU/mL—was observed when LysPB32 (100 μg/mL) was combined with polymyxin B (10.24 mg/mL) after 24 h of incubation at 37 °C, indicating a strong synergistic effect.

Similarly, Shen et al. [24] cloned, expressed, and characterized a recombinant endolysin, rLysJNwz (0–120 μg/mL), encoded by the bacteriophage JNwz02. They evaluated its bacteriostatic effect in combination with EDTA (0.1–25 mM) and polymyxin B (0–1.6 μg/mL) for the biocontrol of *Salmonella enterica* subsp. *enterica* serovar Blukwa (NCTC 8271) (*S. enterica* (NCTC 8271)) in the exponential phase. The optimal EDTA concentration was determined to be 0.5 M, resulting in a 0.8 OD_600_ reduction within 45 min. Polymyxin B’s minimum inhibitory concentration (MIC) decreased from 1.6 to 0.8 µg/mL when combined with 15 µg/mL of rLysJNwz. Notably, rlysJNwz exhibited high stability across temperatures ranging from 4 to 95 °C and pH levels between 5 and 11. Furthermore, the synergy of rLysJNwz (30 or 120 μg/mL) and EDTA (0.5 mM) was evaluated for the biocontrol of *S. enterica* NCTC 8271 (1 × 10^4^ CFU/g or CFU/cm^2^) in eggs and lettuce, incubated at 25 °C for 60 min. The treatment with 30 μg/mL rLysJNwz reduced viable counts by 72.7% in eggs and 85.2% in lettuce, whereas increasing the concentration to 120 μg/mL further reduced viable counts to 86.7% and 86.5%, respectively.

Other studies have also evaluated the use of endolysin delivery systems for the biocontrol of *Gram*-negative bacteria. Bai et al. [99] developed a delivery system using cationic liposomes to facilitate penetration of recombinant endolysin BSP16Lys through the OM barrier of *S. typhimurium* LT2 and *E. coli* (MG1655). The cationic liposomes were formulated using lipid mixtures of dipalmitoylphosphatidylcholine (DPPC), cholesterol (Chol), and hexadecylamine (HDA). The antibacterial activity assay demonstrated reductions of 2.2 log_10_ CFU/mL and 1.6 log_10_ CFU/mL in the number of viable cells for *S. typhimurium* and *E. coli*. The cationic liposomes enhanced bacterial membrane penetration via membrane fusion, driven by strong liposome-cell interactions. This phenomenon is primarily attributed to the anionic nature of *Gram*-negative bacterial membranes, which promotes electrostatic interactions with cationic liposomes [99].

Recently, the lytic activity of endolysins applied in vitro to specific bacterial strains has been evaluated using turbidity reduction assays. Based on this, Xia et al. [105] investigated the lytic activity of the recombinant endolysin LysF23s1 of phage F23s1 in inhibiting *Vibrio parahaemolyticus* (F23). To this end, they applied 20 µmol/L of LysF23s1, decreasing OD from 0.978 to 0.249 within 60 min [106]. The antibacterial capacity of endolysin LysVpKK5, encoded by phage VpKK5, was evaluated against three strains of *Vibrio parahaemolyticus* in seawater at an OD_600_ of 1.2 mixed with 2.5 µg/mL of LysVpKK5. The endolysin exhibited reduced antibacterial activity with increasing NaCl concentration (>0.5 M), likely due to the impact of high ionic strength on electrostatic interactions with the cell wall. These findings suggest that, although this endolysin originates from a marine phage, its natural activity is intracellular; therefore, there is not necessarily a direct correlation between the environmental conditions of phage survival and the optimal conditions of enzymatic activity.

Endolysins have demonstrated potential as agents for the biocontrol of *Gram*-negative bacteria by applying genetic and protein engineering to obtain modified enzymes. Additionally, their efficacy is enhanced by synergistic and additive effects when combined with OMP agents, and other bactericidal compounds, such as antibiotics. Moreover, encapsulating endolysins as an alternative delivery method with membrane-penetration properties is a promising strategy for food application. Encapsulation techniques using liposomes and nanoparticles have demonstrated encouraging outcomes, particularly against *Gram*-negative [107]. However, further research is required to determine the efficacy of endolysin encapsulation and its practical application in food matrices. Likewise, additional studies are needed to analyze the synergistic activity of enzymes combined with OMP agents across different food systems, investigating optimal application conditions, including temperature, pH, NaCl concentration, ultraviolet light exposure, treatment duration, and application concentrations.

## 7. Other Studies of Applications of Phage Enzymes in the Biocontrol of Microorganisms

Phage enzymes have been investigated for their potential in controlling biofilms formed by microbial communities embedded in a matrix of polysaccharides and/or proteins, which confers resistance to antibiotics [108]. Zhang et al. [109], in an in vitro study, applied the lysin LysSTG2 from the STG2 phage in combination with Slightly Acidic Hypochlorous Water (SAHW) to disrupt a biofilm of *S. typhimurium* (NBRC 12529). Viable cells were reduced by 4.8 log_10_ CFU/mL after 72 h of treatment with 100 μg/mL of LysSTG2 and 40 mg/L of SAHW. In a similar study on biofilm control, Ning et al. [110] assessed the lytic and antibiofilm activity of recombinant endolysin Lys84 from phage qdsa002 against *S. aureus* (ATCC 43300). The lytic activity was determined by measuring turbidity reduction, applying 2.5 to 20 µM of Lys84 to an initial OD of 1.40. The results showed a reduction in OD to a minimum of 0.24 after 5 h. Regarding antibiofilm activity, nearly all biofilms were successfully removed using 10 µM of Lys84 for 2 h of treatment.

Meanwhile, Wan et al. [111] evaluated the activity and lytic spectrum of endolysins LysPW2 and LysPW4, encoded by phages PW2 and PW4, respectively. Bacterial cells in the logarithmic growth phase (OD_600_ of 0.8 to 1) were resuspended in reaction buffer (20 mM Tris-HCl, pH 8.0). Lysin concentrations of 0.5, 1.0, and 2.5 µM were tested, with LysPW2 demonstrating superior efficacy at all concentrations. In contrast, LysPW4 significantly reduced bacterial counts only at 2.5 µM. Both enzymes exhibited a broad lytic spectrum, with LysPW2 displaying 96% activity and LysPW4 56%, against tested strains of the *Bacillus cereus* group.

## 8. Strengths and Limitations of Using Enzymes

The use of endolysins for biocontrol bacterial contamination in the food industry offers several advantages, including their ability to lyse bacteria, rapid action, and high efficiency—effective against antibiotic-resistant bacteria and bacterial biofilms. Additionally, endolysins have a low probability of inducing bacterial resistance, as no known cases of resistance have been reported to date [23,96,104,112]. They also exhibit a broad host range, with a wider spectrum of action compared to other phages that encode them [16,36].

Another key advantage of phage enzymes is their strong permeability in biofilms, making them a promising alternative for controlling these complex microbial structures on utensils and surfaces in the food industry. Moreover, they can act on inactive and actively growing bacterial cells, with bacterial lysis dependent on lysin concentration [83].

Regarding their stability under environmental conditions, endolysins demonstrate relative stability across a wide range of temperatures (4 to 60 °C), pH levels, and salt concentrations, making them suitable for application in food production. Additionally, their efficiency can be enhanced through synergistic combinations with other treatments [23]. Compared to phages, phage-derived enzymes are recombinant protein-based molecules, facilitating regulatory approval for food preservation applications [52].

Despite these advantages, the use of phage enzymes presents certain limitations. One of the main challenges is their application for controlling *Gram*-negative bacteria, as the OM protects the peptidoglycan layer from endolysins’ activity. This challenge has been partially overcome through synergistic combinations with OMP agents such as EDTA [98]. Another potential limitation is the generation of neutralizing antibodies, as endolysins are exogenous proteins. However, several studies have demonstrated that lysin-specific antibodies do not inhibit their activity in vitro [95].

A further drawback is the decline in endolysin concentration over time, which can compromise shelf-life activity and leave food vulnerable to bacterial regrowth [83]. Additionally, food production processes’ physicochemical characteristics and environmental conditions can affect their activity. In some cases, both phages and their enzymes may become inactive or exhibit reduced lytic activity. Therefore, identifying optimal conditions is essential to ensure maximum efficiency.

Another major limitation is obtaining large quantities of recombinant endolysins for industrial applications. The production and purification of these enzymes at a large scale can be costly and technically challenging, which may hinder their widespread adoption in the food industry. Therefore, strategies to enhance yield and reduce production costs are crucial for their practical implementation.

## 9. Conclusions

Phages and their derived enzymes have emerged as promising alternatives to conventional preservatives and food safety agents, offering highly specific antibacterial activity. When applied throughout the food chain, these biocontrol agents effectively target pathogenic bacteria without disrupting beneficial microorganisms involved in food production processes, such as probiotic bacteria. The existing literature provides substantial evidence supporting the efficacy of phage enzymes in controlling foodborne pathogens. However, there remains a significant gap in research regarding the encapsulation of endolysins and the extension of their activity over time. Further studies are necessary to evaluate their effectiveness in real-world outbreak scenarios, which would enhance their application across various food matrices during manufacturing and storage.

Due to their ability to degrade peptidoglycan, phage enzymes have proven particularly effective against *Gram*-positive bacteria where the peptidoglycan layer is exposed to the environment. This structural characteristic makes them highly susceptible to enzymatic degradation. However, the application of endolysins against *Gram*-negative bacteria remains challenging due to the presence of an OM that shields the peptidoglycan layer from external agents. To overcome this limitation, synergistic approaches combining endolysins with other antimicrobial agents—such as OMP (organic acids) and HHP treatments—have been developed, enabling endolysins to penetrate the OM of *Gram*-negative bacteria such as *Salmonella*, *Escherichia coli*, and *Pseudomonas*. Additionally, the design of chimeric proteins that integrate endolysins with cationic peptides has demonstrated enhanced activity against *Gram*-negative bacteria, further expanding their potential as biocontrol agents in the food industry.

## Figures and Tables

**Table 1:** Application studies of enzymes in the control of pathogenic bacteria.

Phage	Endolysin	Bacterium	Food	Conditions of Application	Results	Appointment
phiLdb	Lysdb	*S. aureus*	Pasteurized milk in cheese making	Cell conc.: 10^4^ CFU/mL Salt: 2.5% Conditions: 10 °C for 6 weeks	Cell count: Control: 2 × 10^9^ CFU/mL (raw milk) and 3.9 × 10^7^ CFU/g (cheese). Sample with Lysdb in cheese: 2.7 × 10^3^ CFU/g	[60]
PBC4	LysPBC4	*Bacillus cereus*	Turbidity reduction assay (in vitro)	Endolysin conc.: 0.4 μM. Cell conc.: OD_600_: 1.0 Conditions: 37 °C, Tris-HCl 20 mM, pH 8.0	Reduced OD_600_: 1.0 to 0.1 with LysPBC4, after 30 min.	[61]
PBC1	LysPBC1 LysPBC1_EAD	*B. cereus*, *Bacillus subtilis*	Turbidity reduction assay (in vitro)	Endolysin conc.: 0.4 μM. Cell conc.: OD_600_: 1.2 (*B. cereus*) and 1.0 (*B. subtilis*) Conditions: 37 °C, Tris-HCl 20 mM, pH 8.0	In *B. cereus*, OD_600_ decreased 1.2 to 0.2 with LysPBC1 and to 0.7 with LYSPBC1_EAD in 30 min. In *B. subtilis*, OD_600_ decreased from 0.9 to 0.6 with LysPBC1 and to 0.4 with LysPBC1_EAD in 30 min.	[62]
vB_SauS-phiIPLA88	LysH5	*S. aureus* and *S. epidermidis*	In vitro assay against biofilms with 24 h of formation	Endolysin conc.: 0.15 µM, Cell conc.: 10^6^ CFU/mL Additional substances: lysostaphin: 0.2 µM or Sodium phosphate buffer (50 mM, pH 7): 200 µL Conditions: TSB, 6 h at 37 °C	Reduction of sessile cells from 1 to 3 log_10_ CFU/well	[63]
B4	LysB4	*B. cereus*, *B. subtilis*, *L. monocytogenes*. *E. coli*, *P. aeruginosa*, *Cronobacter sakazakii*, *Salmonella* and *Shigella*	Turbidity reduction assay (in vitro)	Endolysin conc.: 0.05 µg/µL Cell conc.: OD_600_: 0.8–1.0 Additional substances: 0.1 M EDTA for *Gram*-negative bacteria.	70% lytic activity in *Gram*-negative, 100% in *B. cereus* and *B*. *subtilis*. Optimal pH: 8–10 Optimal temperature: 50 °C NaCl: 0–200 mM	[64]
FWLLm3	LysZ5	*L. monocytogenes*	Soy milk	Endolysin conc.: 40 U/mL Cell conc.: 10^4^ CFU/mL Conditions: 4 °C for 3 h	Reduction: 5 log_10_ CFU/mL, after 3 h incubation at 4 °C	[65]

## Data Availability

Data availability is not applicable to this article as no new data were created or analyzed in this study.
